# Altered small-world properties of gray matter networks in breast cancer

**DOI:** 10.1186/1471-2377-12-28

**Published:** 2012-05-28

**Authors:** S M Hadi Hosseini, Della Koovakkattu, Shelli R Kesler

**Affiliations:** 1 Department of Psychiatry and Behavioral Sciences, Stanford University School of Medicine, 401 Quarry Road, MC5795, Stanford, CA, 94305-5795, USA; 2 Stanford Cancer Center, Palo Alto, CA, 94304, USA

## Abstract

**Background:**

Breast cancer survivors, particularly those treated with chemotherapy, are at significantly increased risk for long-term cognitive and neurobiologic impairments. These deficits tend to involve skills that are subserved by distributed brain networks. Additionally, neuroimaging studies have shown a diffuse pattern of brain structure changes in chemotherapy-treated breast cancer survivors that might impact large-scale brain networks.

**Methods:**

We therefore applied graph theoretical analysis to compare the gray matter structural networks of female breast cancer survivors with a history of chemotherapy treatment and healthy age and education matched female controls.

**Results:**

Results revealed reduced clustering coefficient and small-world index in the brain network of the breast cancer patients across a range of network densities. In addition, the network of the breast cancer group had less highly interactive nodes and reduced degree/centrality in the frontotemporal regions compared to controls, which may help explain the common impairments of memory and executive functioning among these patients.

**Conclusions:**

These results suggest that breast cancer and chemotherapy may decrease regional connectivity as well as global network organization and integration, reducing efficiency of the network. To our knowledge, this is the first report of altered large-scale brain networks associated with breast cancer and chemotherapy.

## Background

Breast cancer is one of the most frequently diagnosed cancers and the leading cause of cancer death among females, accounting for 23% of the total cancer cases [1]. In the last decade, several neuropsychological studies have shown the negative influence of breast cancer and chemotherapy on various cognitive skills with executive function and memory impairments being the most common [2,3]. These deficits have been reported both prior and following chemotherapy with evidence showing increased and/or more severe cognitive changes in breast cancer patients treated with chemotherapy [4-9]. Neuroimaging studies corroborate these findings by showing changes in both brain structure and function associated with breast cancer and chemotherapy [10]. However, it is currently unknown whether breast cancer and chemotherapy affect large-scale brain networks.

There are several lines of evidence suggesting that breast cancer may negatively impact whole brain networks. First, while the mechanisms by which breast cancer and its treatments affect cognitive function are largely unknown, possible candidates include neurotoxic effects of chemotherapy, oxidative damage, cytokine dysregulation and individual variation in genes related to neural repair and/or plasticity [2,3,11,12]. These candidate mechanisms are likely to have diffuse effects on brain structure. Second, neuroimaging studies indicate that breast cancer survivors show altered brain structure, which would disrupt large-scale networks [13]. Specifically, these patients demonstrate reduced gray matter in bilateral frontal, temporal, cerebellar, thalamic, and cingulate regions as well as decreased white matter integrity in corpus callosum, frontal, and temporal white matter tracts [14-17]. Third, the specific cognitive domains that tend to be most commonly impaired in breast cancer involve executive functions and memory, as noted above. These skills are known to be subserved by distributed, integrated neural networks [18]. Finally, cognitive impairment following breast cancer often tends to be quite subtle [2], potentially suggesting a more diffuse brain injury. In the current study, we investigated whether breast cancer and chemotherapy are associated with alterations in large-scale structural brain networks.

Recent graph-theoretical analyses have consistently shown that brain structural networks in healthy individuals have small-world characteristics [19]; an architecture that has dense local clustering of connections between neighboring nodes with short path length between any pair of nodes due to the existence of relatively few long-range connections [20]. These characteristics, shared by various biological systems, reflect a network that is simultaneously highly segregated and integrated and allows for higher, more efficient rates of information processing and learning than random networks [21].

Since small-world characteristics were described quantitatively for brain structural networks, there have been multiple graph-theoretical studies seeking to assess the structural correlation networks constructed from regional gray matter volume, cortical thickness and surface area [12,20,22,23]. The unique feature of graph-theoretical analysis, compared with the more traditional univariate neuroimaging approaches, is that it can directly test the differences in topological parameters of the brain network such as small-worldness, highly connected hubs and regional network parameters. Whereas univariate neuroimaging approaches have typically shown limited correlations with cognitive function and dysfunction, network parameters may provide a more robust model of cognitive status [24,25]. Recent graph-theoretical studies have illustrated an alteration of arrangements in structural correlation networks associated with normal aging, multiple sclerosis, Alzheimer’s disease, schizophrenia and epilepsy [26-30].

In the present study, we applied graph theoretical analyses to compare magnetic resonance imaging (MRI)-based gray matter correlation networks of female breast cancer patients treated with chemotherapy and female healthy controls. Considering the lines of evidence regarding a diffuse pattern of gray matter atrophy in breast cancer patients, we hypothesized that such alterations should be reflected in small-world characteristics of the brain structural correlation network. We also examined the between group differences in highly connected hubs as well as in regional network measures such as node betweenness and degree.

## Methods

### Participants

Thirty-seven female primary breast cancer survivors (BC) age 43-67 years who were treated with systemic chemotherapy (mean time since treatment = 4.5 ± 3.4 years) and 38 age-matched healthy controls (CON) participated in the study. BC survivors were recruited via the Army of Women (http://www.armyofwomen.org/), community-based BC support groups and local media advertisements. Healthy controls were recruited via the Army of Women and local media advertisements. Groups were matched for age, education, premorbid cognitive functioning and minority status although there were significantly more post-menopausal women in the BC group (Table 1). Premorbid cognitive functioning was measured using the Information subtest of the Wechsler Adult Intelligence Scale 4th Edition. This subtest measures fund of general knowledge and is believed to be influenced by previous experience and quality of education, making it a good estimate of premorbid ability [31]. Participants in the BC group were free from disease and had no history of relapse or recurrence at the time of evaluation. Chemotherapy regimens included AC (Doxorubicin, Cyclophosphamide) (N = 9), ACT (Doxorubicin, Cyclophosphamide, Paclitaxel) (N = 16), ACF (Doxorubicin, Cyclophosphamide, 5-Fluorouracil) (N = 2), CT (Cyclophosphamide, Paclitaxel) (N = 6), CMF (Cyclophosphamide, Methotrexate, 5-Fluorouracil) (N = 2), CTF (Cyclophosphamide, Paclitaxel, 5-Fluorouracil) (N = 1) and AC + CMF (N = 1). Additionally, 15 women were treated with tamoxifen and 25 received radiation therapy. None of the BC patients had cerebral metastasis or had been treated for that. Participants were excluded for neurologic, psychiatric or medical conditions known to affect cognitive function (e.g. learning disability, traumatic brain injury, chronic depression) as well as any MRI contraindications (e.g. non-MRI safe implants). This study was approved by the Stanford University Institutional Review Board and all participants provided informed consent.

**Table 1:** Demographic data for the breast cancer and healthy control groups

	BC (N = 37)	CON (N = 38)	t or Chi Sq.	p-value
age	54.2 (6.1)	55.5 (9.0)	.737	.46
education	16 (2.8)	17 (2.6)	1.61	.11
minority status	8.6%	10.5%	2.27	.69
post menopause	86.7%	55.9%	7.24	.007
premorbid cognitive status*	13.0 (6.0)	13.8 (2.7)	.774	.44

*measured using the Information subtest of the Wechsler Adult Intelligence Scale 4th Edition.

Data are shown as mean (standard deviation) except where noted.

### MRI data acquisition and preprocessing

MRI scanning was performed on a GE Discovery MR750 3.0 Tesla whole body scanner (GE Medical Systems, Milwaukee, WI). High-resolution T1-weighted images were acquired with 3D spoiled gradient echo pulse sequence using the following parameters: TR = 8.5 ms, TE = 3.396, TI = 400 ms, flip angle = 15, FOV = 220 mm, number of excitation =1, acquisition matrix = 256 x 192. Totally, 124 contiguous coronal slices were with in-plane resolution of 0.859 mm x 0.859 mm.

Image preprocessing was performed using Statistical Parametric Mapping 8 (SPM8; Wellcome Department of Cognitive Neurology, London, UK; http://www.fil.ion.ucl.ac.uk/spm/). The images were initially segmented into gray matter (GM), white matter, and cerebrospinal fluid images based on the ICBM Tissue Probabilistic Maps (http://www.loni.ucla.edu/ICBM/ICBM TissueProb.html). A study-specific a priori probability map of GM was created from the modulated spatially normalized segmented GM images using the Template-O-Matic (TOM8) toolbox [32]. Next, the custom priors were affine-registered to the standard Montreal Neurological Institute (MNI) space.

Voxel-based morphometry (VBM) preprocessing steps were performed using the VBM8 toolbox for SPM8 (http://dbm.neuro.uni-jena.de/vbm/) and this involved segmentation of MR images into GM tissue segments using the standard unified segmented and non-linearly warping of the tissue segments to the GM study-specific customized template model [33]. Images were then modulated to ensure that relative volumes of GM were preserved following the spatial normalization procedure. Sample homogeneity was checked to identify any outliers in the study population. Data for one participant in the BC group were excluded from the analysis because of a covariance below 2 SD and confirmation of visual motion artifact.

### ROI extraction

There are different nodal definition methods in brain network analysis. While the results might be affected by the choice of parcellation scheme, recent evidence showed that the results of between-group comparison remain intact regardless of the applied parcellation scheme [34]. We generated 90 cortical and subcortical regions of interest (ROIs), excluding the cerebellum, from the Automated Anatomical Labeling (AAL) atlas using the WFU PickAtlas Toolbox [35]. The ROIs were identical to those used in a previous graph analysis study of typical brain development by Fan and colleagues [22]. These AAL ROIs were resliced to the same dimension as that of tissue segmented images obtained from the VBM preprocessing step. The ROIs were subsequently used to mask the individual modulated, normalized GM images and extract the average volume within each ROI using the REX toolbox (http://web.mit.edu/swg/software.htm). A linear regression analysis was performed at every ROI to remove the effects of age and total brain volume. The residuals of this regression were then substituted for the raw ROI volume values [22,27,36].

### Construction of structural correlation network

The extracted residual volumes of all 90 anatomical ROIs were used for construction of structural correlation networks. For each group, a 90 × 90 association matrix *R* was generated with each entry *r*_*ij*_ defined as the Pearson correlation coefficient between residual volumes of regions *i* and *j*, across subjects. From each association matrix, a binary adjacency matrix *A* was derived where *a*_*ij*_ was considered 1 if *r*_*ij*_ was greater than a specific threshold and zero otherwise. The diagonal elements of the constructed association matrix were also set to zero. The resultant adjacency matrix represented a binary undirected graph *G* in which regions *i* and *j* were connected if *g*_*ij*_ was unity. Therefore, a graph was constructed with *N* = 90 nodes (anatomical ROIs), with a network degree of *E* equal to number of edges (links), and a network density (cost) of D = E/[(N x (N-1))/2] representing the fraction of present connections to all possible connections. Since thresholding the association matrices of different groups at an absolute threshold results in networks with a different number of nodes (and degrees) that might influence the network measures and reduce interpretation of between group results [37], two approaches were implemented for thresholding the constructed association matrices based on previous studies [26-28]: (1) Thresholding the constructed association matrices at a minimum network density in which all nodes become fully connected in the brain networks of both groups; (2) Thresholding the constructed association matrices at a range of network densities and comparing the network topologies across that range.

#### Network analysis

##### Small-worldness

The small-worldness of a complex network, as described above, has two key metrics: the clustering coefficient *C* and the characteristic path length *L* of the network. The clustering coefficient of a node is a measure of the number of edges that exist between its nearest neighbors. The clustering coefficient of a network is thus the average of clustering coefficients across nodes and is a measure of network segregation. The characteristic path length of a network is the average shortest path length between all pairs of nodes in the network and is the most commonly used measure of network integration [38]. To evaluate the topology of the brain network, these parameters must be compared to the corresponding mean values of a random graph with the same number of nodes, total edges, and degree distribution as the network of interest [39,40]. Thus, we obtained the small-worldness index of a network as [C/C_rand_/[L/L_rand_ where *C*_*rand*_ and *L*_*rand*_ are the mean clustering coefficient and the characteristic path length of the random network [20]. In a small-world network, the clustering coefficient is significantly higher than that of random networks (*C/C*_*rand*_ ratio greater than 1) while the characteristic path length is comparable to random networks (*L/L*_*rand*_ ratio close to 1).

##### Regional network measures

We also investigated the nodal characteristics of the constructed structural networks to identify differences in regional network measures between groups. Nodal betweenness centrality and nodal degree were calculated for each of the anatomical ROIs for the networks thresholded at minimum density with full-connectivity. Nodal betweenness centrality is defined as the fraction of all shortest paths in the network that pass through a given node and is used to detect important anatomical or functional connections. Nodes that bridge disparate parts of the network have a high betweenness centrality [38]. On the other hand, nodal degree is defined as the number of connections that a node has with the rest of the network and is considered a measure of interaction of a node, structurally or functionally, with the network.

##### Network hubs

Hubs are crucial components for efficient communication in a network. Hubs are not only considered as important regulators of information flow but also play a key role in network resilience to insult [38]. We considered a node as a hub if its betweenness centrality was at least 2SD higher than mean network centrality [26].

##### Comparing correlation strengths between groups

To test the significance of differences in overall inter-regional correlation of gray matter volume between networks, the correlation coefficients were converted to *z* values using Fisher’s r-to-z transform. This transformation resulted in values that were approximately normally distributed [28]. Then, a two sample *t*-test was used to test the significance of the difference in mean overall correlation between groups.

##### Comparing network measures between groups

In order to test the statistical significance of the between-group differences in network topology and regional network measures, a non-parametric permutation test with 1000 repetitions was used [26,28]. In each repetition, the calculated residual volumes of each participant were randomly reassigned to one of the two groups so that each randomized group had the same number of subjects as the original groups. Then, an association matrix was obtained for each randomized group. The binary adjacency matrices were then estimated by thresholding the association matrices at a range of network densities. The network measures were then calculated for all the networks at each density. The differences in network measures between randomized groups were then calculated resulting in a permutation distribution of difference under the null hypothesis. The actual between-group difference in network measures was then placed in the corresponding permutation distribution and a two-tailed p-value was calculated based on its percentile position [27].

We used the Brain Connectivity Toolbox [38] for quantification of network measures in addition to in-house software, Graph Analysis Toolbox (http://nnl.stanford.edu/Tools.html), for comparing the structural networks. Brain Net Viewer (http://www.nitrc.org/projects/bnv/) was used for visualization of the graphs.

It should be noted that we could not statistically explore the relationship between cognitive-behavioral measures and the extracted network properties. It is because the extracted network measures are group-specific and can not be quantified for individuals. Therefore, we could not perform statistical analysis (e.g. correlation analysis) to relate network properties and cognitive-behavioral outcomes.

## Results

### Comparing networks at minimum density with full-connectivity

**Figure 1:**
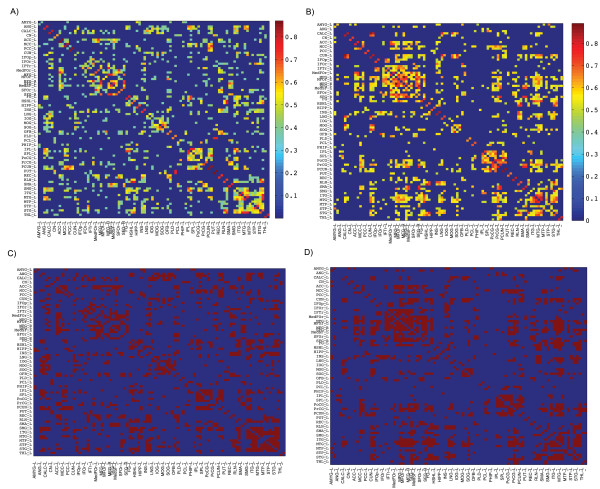
**Association and binary adjacency matrices; association matrices for (A) BC and (B) CON groups, binary adjacency matrices for (C) BC and (D) CON groups (connected regions are shown in red).** The association matrices show the highest connectivity between regions of interest as well as for inter-hemispheric regions.

The minimum network density in which all nodes became fully connected in the structural networks of both groups was 0.184. The association matrices for each group and corresponding binary adjacency matrices thresholded at the minimum density are shown in Figure 1. The correlation network of the BC group showed lower overall correlation strength than the CON group (*t = 5.9, P <0.001*). Topological measures for the networks of both groups thresholded at the density of 0.184 are given in Table 2. In both groups, the normalized characteristic path length of the network was close to 1 and the normalized clustering coefficient was greater than 1 which results in a small-worldness parameter of greater than 1. Thus, consistent with previous studies, the gray-matter correlation network of both groups followed a small-world property. While no significant difference in characteristic path length was observed between networks (*P = 0.22*), the normalized clustering coefficient was significantly higher in CON network (*P < 0.05*). Consequently, the small-worldness parameter was found to be greater in the CON network than in the BC network and the observed difference was marginally significant (*P = 0.08*).

**Table 2:** Network measures at minimum density of 0.184 with full connectivity

	BC	CON	p-value
Density	0.184	0.184	---
Mean clustering coefficient	0.485	0.517	0.16
Characteristic path length	2.11	2.2	0.23
Normalized clustering	2.65	3.06	0.03
Normalized path length	1.15	1.20	0.22
Small-worldness	2.30	2.54	0.08

### Comparing networks across a range of density

**Figure 2:**
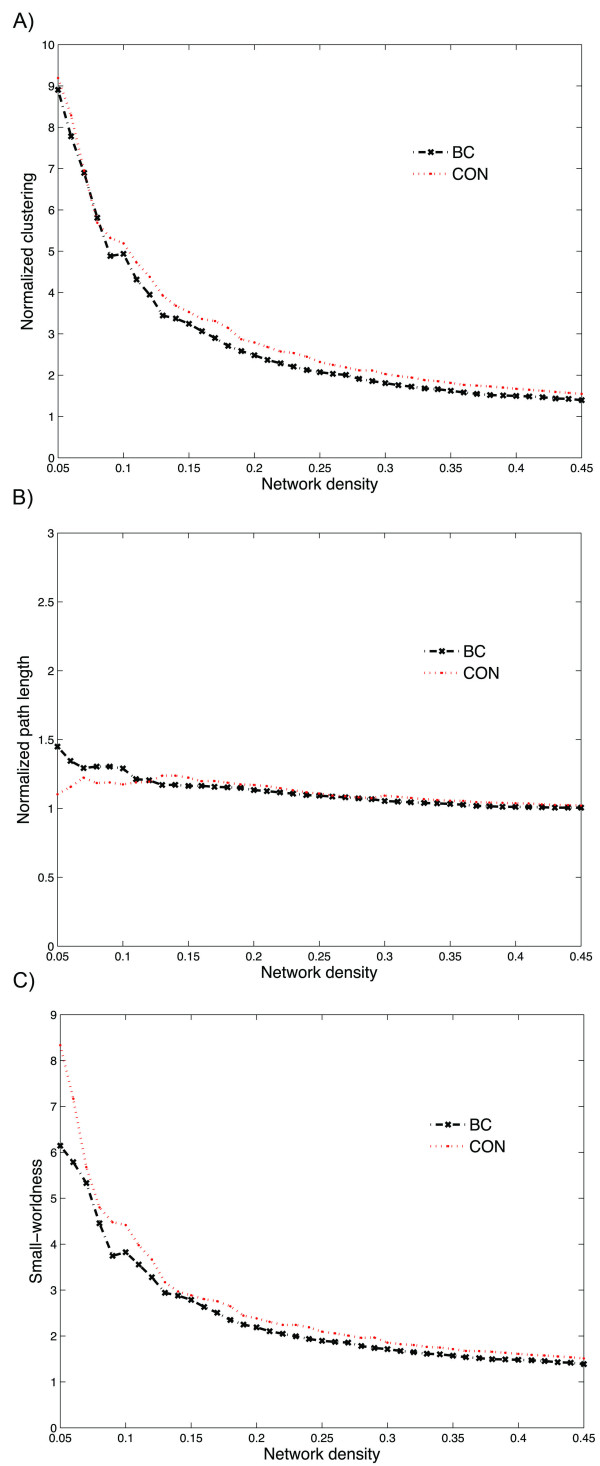
**Global network measures; A) clustering coefficient, B) characteristic path length and C) small-worldness of BC and CON networks.** The figures show that both networks follow a small-world organization across the range of densities; the characteristic path length is close to 1 (for the fully connected network, i.e. density > 0.19) and the clustering is greater than 1 in different densities.

**Figure 3:**
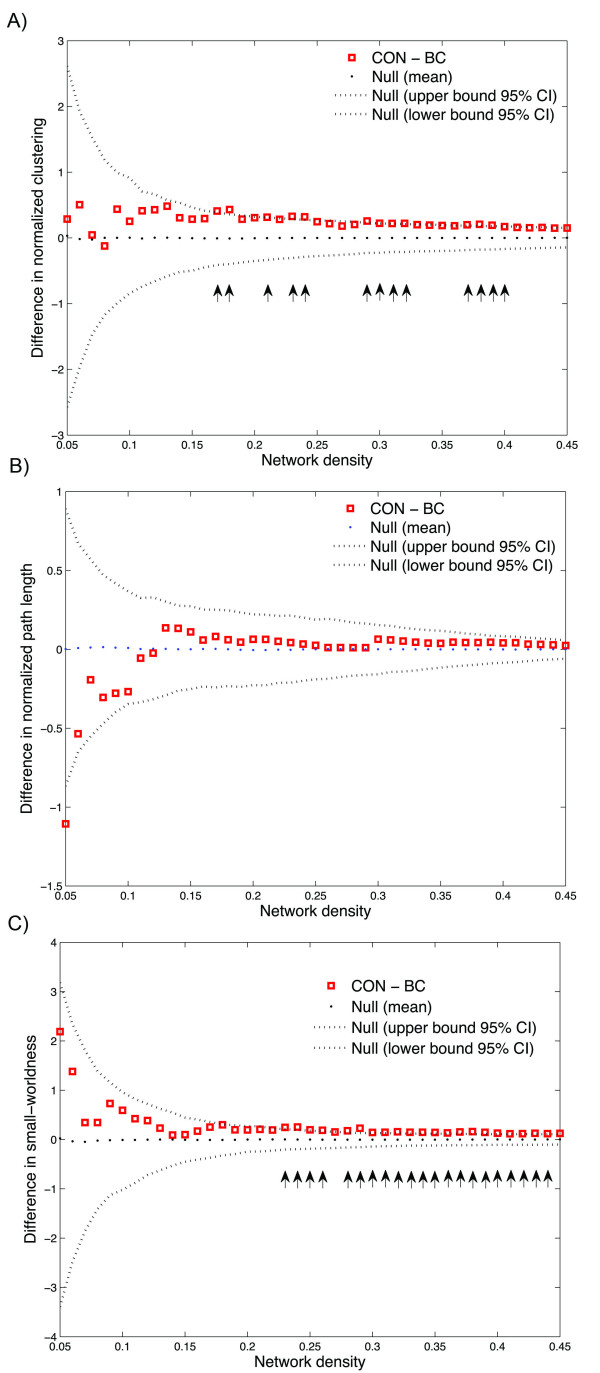
**Between-group differences in global network measures; between-group differences in A) clustering coefficient, B) characteristic path length and C) small-worldness parameter.** The vertical arrows represent the densities where the difference is statistically significant at *P < 0.05*. It shows that while there is no significant difference in network characteristic path lengths between groups (same level of integration), the clustering coefficient is significantly higher (more segregated network) in the CON network in various densities resulting in higher small-worldness compared to the BC group across a range of densities. Squares represent control minus BC group measure, dashed lines show the 95% confidence interval and the dotted line shows the mean for the random graph distribution.

In order to investigate changes in the network topology as a function of network density (cost), we thresholded the constructed association matrices at a range of network densities. Changes in network parameters are depicted in Figure 2. The figures show that both networks follow a small-world organization across the range of densities. The results of comparison of the two networks as a function of network density are shown in Figure 3. While there was no significant difference in network characteristic path lengths between groups (same level of integration), the clustering coefficient was significantly higher (more segregated network) in the CON network in various densities resulting in higher small-worldness compared to the BC group across a range of densities.

Below the minimum density of full connectivity (0.184), the BC network fragments sooner that the CON network and thus the normalized path length of the BC network increases relatively faster compared with that of CON network. This results in a relatively large drop in the difference in normalized path length between groups in densities below 0.184.

### Regional network measures

**Figure 4:**
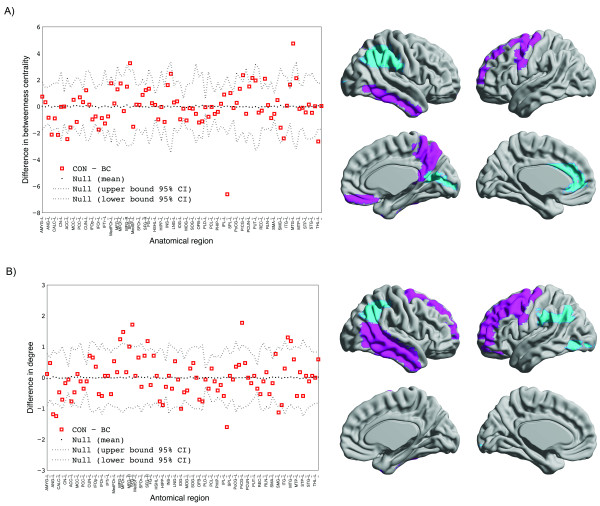
**Difference in regional network characteristics relative to random networks; A) Controls showed greater network betweenness centrality in left SFG, left PrCG, right PCUN, right REC and right ITG while the BC group showed greater betweenness in right ANG, right CALC, left ACC, right IPL, and left THL. B) Controls demonstrated higher network degree in left MFG, left MFOr, bilateral SFG, left PrCG, right ITG and right MTG.** The BC group showed greater degree in bilateral ANG, left IOG, right IPL and left SMG. Squares represent control minus BC group measure, dashed lines show the 95% confidence interval and the dotted line shows the mean for the random graph distribution. Regions that showed significantly higher/lower degree in CON relative to BC are shown in pink/cian color on the ICBM152 brain template. Abbreviations are used as follow: L: left hemisphere; AMYG: amygdala; ANG: angular gyrus; CALC: calcarine fissure; CN: caudate nucleus; ACC: anterior cingulate; MCC: mid-cingulate; PCC: posterior cingulate; CUN: cuneus; IFOp: inferior frontal gyrus, opercular part; IFOr: inferior frontal gyrus, orbital part; IFTr: inferior frontal gyrus, triangular part; MedFOr: medial frontal gyrus, orbital part; MFG: middle frontal gyrus; MFOr: middle frontal gyrus, orbital part; SFG: superior frontal gyrus; MedSF: superior frontal gyrus, medial part; SFOr: superior frontal gyrus, orbital part; FG: fusiform gyrus; HSHL: heschl gyrus; HIPP: hippocampus; INS: insula; LNG: lingual gyrus; IOG: inferior occipital gyrus; MOG: middle occipital gyrus; SOG: superior occipital gyrus; OFB: olfactory cortex; PLD: lenticular nucleus, pallidum; PCL: paracentral lobule; PHIP: parahippocampal gyrus; IPL: inferior parietal lobule; SPL: superior parietal lobule; PoCG: postcentral gyrus; PrCG: precentral gyrus; PCUN: precuneus; PUT: putamen; REC: gyrus rectus; RLN: rolandic operculum; SMA: supplementary motor area; SMG: supramarginal gyrus; ITG: inferior temporal gyrus; MTG: middle temporal gyrus; MTP: middle temporal pole; STP: superior temporal pole; STG: superior temporal gyrus; THL: thalamus.

As shown in Figure 4, the CON group demonstrated higher nodal betweeness/degree in several frontal and temporal regions. The BC group demonstrated higher nodal betweeness/degree primarily in parieto-occipital regions but also in the anterior cingulate and left thalamus.

### Network hubs

**Figure 5:**
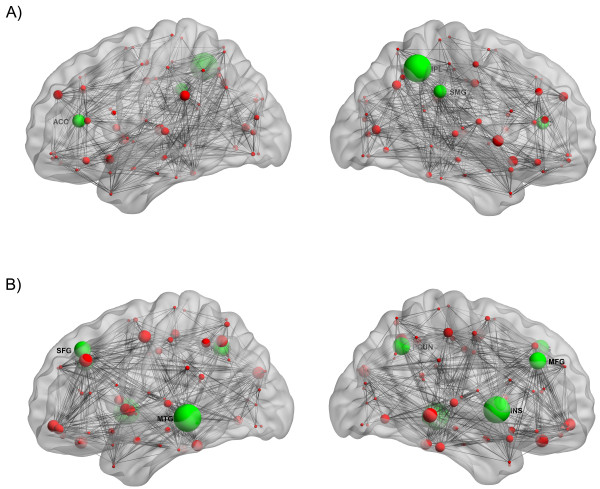
**Network hubs; structural correlation networks and hubs overlaid on ICBM152 brain template for A) BC and B) CON groups.** Grey lines indicate connections and spheres represent regions. The radius of the spheres is proportional to the nodal betweenness. Hubs are shown in green color.

In the BC group, network hubs were identified at left anterior cingulate, right inferior parietal lobule, and right supramarginal gyrus. In CON group, hubs were found in right middle frontal gyrus, left superior frontal gyrus, right insula, right precuneus, and left middle temporal gyrus (Figure 5).

## Discussion

We applied graph theoretical analyses to compare gray matter volume correlation networks of female primary breast cancer (BC) survivors treated with chemotherapy and female healthy controls (CON). We found an alteration in small-world characteristics of the brain structural network in the BC group; an observation that confirms our hypothesis suggesting changes in large-scale brain network properties in BC survivors treated with chemotherapy. To our knowledge, this is the first report of altered large-scale network properties associated with BC and chemotherapy.

### Between-group differences in network topology

While the structural correlation networks of both groups followed a small-world organization, the results revealed a decrease in the small-world index of the BC group compared to controls. The characteristic path lengths of the BC and CON group networks were not different whereas the clustering coefficient was significantly smaller in the BC network than in the CON network. Decreased clustering coefficient and small-world index in the BC group was observed not only at the minimum network density but also over a range of density thresholds.

These findings suggest that the structural correlation networks of BC patients tend to have a more randomized configuration compared to CON group associated with weaker regional connectivity and disrupted global organization [38]. Given that BC tends to be a disease associated with aging, altered network topology could make women with BC more vulnerable to age-related cognitive decline. Recent studies suggest that older BC patients show increased chemotherapy-related cognitive deficits [4,5]. Additionally, this same profile of network topology (i.e. decreased clustering and small-world index) has been associated with Alzheimer’s dementia [41]. The interface between aging, breast cancer and cognitive decline will be a critical area of continued investigation.

The observed lack of densely interconnected neighbors in the network of BC group is also supported by the lower overall correlation strength in the association matrix of BC group compared to CON group. Together, the results of network-level analysis corroborates previous structural neuroimaging findings that have demonstrated a diffuse pattern of atrophy in gray matter volumes in BC patients [15,16] and further suggests an alteration in the coordinated patterns of brain morphology in the structural network of these patients.

One potential mechanism underlying this network-level alteration is white matter damage. There is a large body of evidence suggesting that global gray matter atrophy is associated with focal and global white matter damage due in part to the transection of axons and subsequent retrograde neuronal loss [42,43]. Animal studies show that chemotherapy suppresses neural progenitor cell proliferation responsible for white-matter tract integrity and cortico-cortical connections [44-48]. Thus, the observed network-level alteration in structural correlation network of BC patients might arise from neurotoxic effects of chemotherapy on cortico-cortical connections. This idea is also supported by a recent diffusion weighted imaging study involving chemotherapy-treated BC survivors that reported a diffuse pattern of microstructural white matter damage [14].

### Between-group differences in regional network measures

High degree/betweenness nodes were observed mainly in frontotemporal regions in the CON network and in parietal, cingulate, and occipital regions in the BC network. Nodes with high degree/centrality in the structural network identify regions that are highly interactive and have the potential to participate in a large number of functional interactions [49]. The results indicate that the network of BC group has less highly-interactive nodes in the frontotemporal regions.

Reduction in gray and white matter volumes in frontotemporal regions of patients with BC before and after treatment has been shown in a recent study [15]. A number of functional neuroimaging studies have also reported increased activation in the frontotemporal network in these patients suggesting necessity of recruiting a compensatory neural mechanism to complete certain tasks, or decreased efficiency of these systems [10,50,51]. The observed changes in the centrality of the frontotemporal nodes in the network of BC patients is consistent with these previous results and may explain the impairment of these patients in memory and executive functioning.

There are several mechanisms that could explain the observed change in the distribution of highly influential nodes in BC group with less highly-interactive nodes in the prefrontal and temporal cortices. Certain genetic and immunologic factors have been proposed as important contributors to cognitive decline in patients with BC [3]. For example, apolipoprotein E (APOE) is a complex protein that has an important role in neuronal repair and plasticity after injury [52]. The relationship between this genotype and adverse cognitive performance and reduce mesial temporal (hippocampal) volume has been shown in long-term survivors of BC [53]. Also, cytokines that become dysregulated during cancer and/or chemotherapy treatment have been shown to affect cognitive function in healthy adults [54]. Cytokines have important roles in central nervous system functioning including modulation of dopamine and other neurotransmitters [55] that affect the frontotemporal network [56]. The effects of genotypic variations and cytokine regulation on large-scale networks have not been established and require further investigation.

The BC group had significantly more post-menopausal women than the healthy comparison group. This is expected given that chemotherapy frequently causes early menopause [57] and tamoxifen can increase menopausal symptoms [19], both by blocking or reducing estrogen. Estrogen has a marked effect on brain function, particularly in frontotemporal regions and their concomitant executive and memory functions [58]. Previous studies suggest that tamoxifen, in addition to chemotherapy, can have detrimental effects on cognitive and neurobiologic outcomes in BC survivors [59,60]. The present findings may indicate potential secondary neuroendocrine effects on large-scale brain networks.

### Network hub analysis

There was a difference in the number and distribution of hubs between the BC and CON groups. We found six network hubs in the CON group mainly across frontal and temporal regions compared to three hubs in the BC group primarily in parietal and cingulate areas. The identified hubs in the CON group are consistent with the results of previous graph-theoretical analysis involving healthy adults [26,28]. The present findings indicate that the network of BC patients does not show expected prefrontal and temporal hubs suggesting network alterations involving these regions, again which are critical for the executive function and memory skills commonly disrupted in BC.

Additionally, the number of identified hubs in the BC network was half the number of hubs in the CON network. Since hubs are crucial components for efficient communication across the network, this observation suggests a less efficient information transfer in the network of BC patients. These results are consistent with our previous findings showing that BC survivors can often perform similarly to controls on cognitive tasks but require significantly more neural resources to do so [50]. Standardized neurocognitive tests may lack sensitivity to these subtle changes [2] as most were designed to assess specific cognitive skills. Based on the present findings, measures of reaction time or parallel processing should be included in evaluations of BC-related cognitive outcome. Quantitative process measures might also be more effective as these assessments examine the efficiency of the patient’s performance strategies in addition to performance accuracy [61].

This study has several limitations. First, this was a cross-sectional study and therefore, we could not directly test the dissociated effects of cancer and chemotherapy on network measures. Future studies can address this issue through longitudinal evaluations of network measures in patients with BC. In addition, future studies might investigate whether the alteration in gray matter network is due to any cancer (and not just breast cancer). It is also important to dissociate between cancer-related physiological changes and cancer effects on mental states. Second, the network measures were identified by calculating the correlations of gray matter volume across subjects. While this methodology is common for investigating between-group differences in brain structural network, it lacks the ability to explore the individual differences in network parameters. Specifically, we could not explore the effect of treatment period, regimen, age or other individual differences on network parameters or investigate the effects of altered network parameters on cognitive-behavioral outcome.

## Conclusions

Despite these limitations, these findings increase our understanding of BC and chemotherapy-related cognitive impairment by demonstrating alterations in specific network properties. Survivors of BC treated with chemotherapy showed decreased regional connectivity (clustering coefficient) and global network organization (small-worldness) and integration (hubs) suggesting reduced robustness and efficiency of the network. A pattern of frontotemporal abnormality was also noted. These results contribute novel insights regarding the neurobiologic mechanisms underlying cognitive deficits in these patients and highlight critical areas for future research related to the potential vulnerability of BC patients to the neurologic effects of aging and other forms of structural damage.

## Competing interests

None.

## Authors’ contributions

HH wrote code for the analysis toolbox, analyzed the graph theory data and co-wrote the manuscript. DK assisted with volumetric data acquisition and analyses and co-wrote the manuscript. SK conceived and directed development of the analysis toolbox, designed and supervised the study, conducted demographic and behavioral data analyses and co-wrote the manuscript.

## Pre-publication history

The pre-publication history for this paper can be accessed here:

http://www.biomedcentral.com/1471-2377/12/28/prepub

## References

[B1] Jemal A Bray F Center MM Ferlay J Ward E Forman D Global cancer statistics 2011 a cancer journal for clinicians, CA 10.3322/caac.20107 21296855

[B2] Vardy J Cognitive function in breast cancer survivors Cancer Treat Res 2009 151 387 419 10.1007/978-0-387-75115-3_24 19593525

[B3] Janelsins MC Kohli S Mohile SG Usuki K Ahles TA Morrow GR An update on cancer- and chemotherapy-related cognitive dysfunction: current status Semin Oncol 2011 38 431 438 10.1053/j.seminoncol.2011.03.014 21600374 PMC3120018

[B4] Kesler SR Kent J O'Hara R Prefrontal cortex and executive function impairments in primary breast cancer Arch Neurol 2011 68 1447 1453 10.1001/archneurol.2011.245 22084128 PMC3239218

[B5] Ahles TA Saykin AJ McDonald BC Li Y Furstenberg CT Hanscom BS Mulrooney TJ Schwartz GN Kaufman PA Longitudinal Assessment of Cognitive Changes Associated With Adjuvant Treatment for Breast Cancer: Impact of Age and Cognitive Reserve J Clin Oncol 2010 28 4434 4440 10.1200/JCO.2009.27.0827 20837957 PMC2988635

[B6] Quesnel C Savard J Ivers H Cognitive impairments associated with breast cancer treatments: results from a longitudinal study Breast Cancer Res Treat 2009 116 113 123 10.1007/s10549-008-0114-2 18629633

[B7] Schagen SB Muller MJ Boogerd W Mellenbergh GJ van Dam FS Change in cognitive function after chemotherapy: a prospective longitudinal study in breast cancer patients J Natl Cancer Inst 2006 98 1742 1745 10.1093/jnci/djj470 17148777

[B8] Jansen CE Cooper BA Dodd MJ 2010 A prospective longitudinal study of chemotherapy-induced cognitive changes in breast cancer patients. Support Care Cancer, Miaskowski CA 10.1007/s00520-010-0997-4 20820813

[B9] Wefel JS Saleeba AK Buzdar AU Meyers CA Acute and late onset cognitive dysfunction associated with chemotherapy in women with breast cancer Cancer 2010 116 3348 3356 10.1002/cncr.25098 20564075

[B10] de Ruiter MB Reneman L Boogerd W Veltman DJ van Dam FS Nederveen AJ Boven E Schagen SB Cerebral hyporesponsiveness and cognitive impairment 10 years after chemotherapy for breast cancer Hum Brain Mapp 2011 32 1206 1219 10.1002/hbm.21102 20669165 PMC6869999

[B11] Wefel JS Witgert ME Meyers CA Neuropsychological sequelae of non-central nervous system cancer and cancer therapy Neuropsychol Rev 2008 18 121 131 10.1007/s11065-008-9058-x 18415683

[B12] Ahles TA Saykin AJ Candidate mechanisms for chemotherapy-induced cognitive changes Nat Rev Cancer 2007 7 192 201 10.1038/nrc2073 17318212 PMC3329763

[B13] Seeley WW Crawford RK Zhou J Miller BL Greicius MD Neurodegenerative diseases target large-scale human brain networks Neuron 2009 62 42 52 10.1016/j.neuron.2009.03.024 19376066 PMC2691647

[B14] Deprez S Amant F Yigit R Porke K Verhoeven J Van den Stock J Smeets A Christiaens MR Leemans A Van Hecke W Chemotherapy-induced structural changes in cerebral white matter and its correlation with impaired cognitive functioning in breast cancer patients Hum Brain Mapp 2011 32 480 493 10.1002/hbm.21033 20725909 PMC6870393

[B15] McDonald BC Conroy SK Ahles TA West JD Saykin AJ Gray matter reduction associated with systemic chemotherapy for breast cancer: a prospective MRI study Breast Cancer Res Treat 2010 123 819 828 10.1007/s10549-010-1088-4 20690040 PMC3661415

[B16] Inagaki M Yoshikawa E Matsuoka Y Sugawara Y Nakano T Akechi T Wada N Imoto S Murakami K Uchitomi Y Smaller regional volumes of brain gray and white matter demonstrated in breast cancer survivors exposed to adjuvant chemotherapy Cancer 2007 109 146 156 10.1002/cncr.22368 17131349

[B17] Abraham J Haut M Moran M Filburn S Lemiuex S Kuwabara H Adjuvant chemotherapy for breast cancer: effects on cerebral white matter seen in diffusion tensor imaging Clin Breast Cancer 2008 8 88 91 10.3816/CBC.2008.n.007 18501063

[B18] Leh SE Petrides M Strafella AP The Neural Circuitry of Executive Functions in Healthy Subjects and Parkinson's Disease Neuropsychopharmacology 2010 35 70 85 10.1038/npp.2009.88 19657332 PMC3055448

[B19] Bakkum-Gamez JN Laughlin SK Jensen JR Akogyeram CO Pruthi S Challenges in the Gynecologic Care of Premenopausal Women With Breast Cancer Mayo Clin Proc 2011 86 229 240 10.4065/mcp.2010.0794 21307388 PMC3046944

[B20] Bassett DS Bullmore E Small-world brain networks Neuroscientist 2006 12 512 523 10.1177/1073858406293182 17079517

[B21] Simard D Nadeau L Fastest learning in small-world neural networks Physics Letters A 2005 336 8 15 10.1016/j.physleta.2004.12.078

[B22] Fan Y Shi F Smith JK Lin W Gilmore JH Shen D Brain anatomical networks in early human brain development NeuroImage 2011 54 1862 1871 10.1016/j.neuroimage.2010.07.025 20650319 PMC3023885

[B23] Chen ZJ He Y Rosa-Neto P Germann J Evans AC Revealing modular architecture of human brain structural networks by using cortical thickness from MRI Cerebral cortex 2008 18 2374 2381 10.1093/cercor/bhn003 18267952 PMC2733312

[B24] Petrella JR Use of graph theory to evaluate brain networks: a clinical tool for a small world? Radiology 2011 259 317 320 10.1148/radiol.11110380 21502388

[B25] Sporns O Chialvo DR Kaiser M Hilgetag CC Organization, development and function of complex brain networks Trends Cogn Sci 2004 8 418 425 10.1016/j.tics.2004.07.008 15350243

[B26] Bassett DS Bullmore E Verchinski BA Mattay VS Weinberger DR Meyer-Lindenberg A Hierarchical organization of human cortical networks in health and schizophrenia J Neurosci 2008 28 9239 9248 10.1523/JNEUROSCI.1929-08.2008 18784304 PMC2878961

[B27] Bernhardt BC Chen Z He Y Evans AC Bernasconi N Graph-Theoretical Analysis Reveals Disrupted Small-World Organization of Cortical Thickness Correlation Networks in Temporal Lobe Epilepsy Cerebral Cortex 2011 21 9 2147 2157 10.1093/cercor/bhq291 21330467

[B28] He Y Chen Z Evans A Structural insights into aberrant topological patterns of large-scale cortical networks in Alzheimer's disease J Neurosci 2008 28 4756 4766 10.1523/JNEUROSCI.0141-08.2008 18448652 PMC6670444

[B29] Wu K Taki Y Sato K Kinomura S Goto R Okada K Kawashima R He Y Evans AC Fukuda H Age-related changes in topological organization of structural brain networks in healthy individuals Hum Brain Mapp 2011 33 3 552 568 21391279 10.1002/hbm.21232 PMC6870030

[B30] He Y Dagher A Chen Z Charil A Zijdenbos A Worsley K Evans A Impaired small-world efficiency in structural cortical networks in multiple sclerosis associated with white matter lesion load Brain 2009 132 3366 3379 10.1093/brain/awp089 19439423 PMC2792366

[B31] Lowe DA Rogers SA Estimating Premorbid Intelligence among Older Adults: The Utility of the AMNART J Aging Res 2011 2011 428132 21629758 10.4061/2011/428132 PMC3100635

[B32] Wilke M Holland S Altaye M Gaser C Template-O-Matic: A toolbox for creating customized pediatric templates NeuroImage 2008 41 903 913 10.1016/j.neuroimage.2008.02.056 18424084

[B33] Ashburner J Friston KJ Unified segmentation NeuroImage 2005 26 839 851 10.1016/j.neuroimage.2005.02.018 15955494

[B34] Zalesky A Fornito A Harding IH Cocchi L Yucel M Pantelis C Bullmore ET Whole-brain anatomical networks: does the choice of nodes matter? NeuroImage 2010 50 970 983 10.1016/j.neuroimage.2009.12.027 20035887

[B35] Tzourio-Mazoyer N Landeau B Papathanassiou D Crivello F Etard O Delcroix N Mazoyer B Joliot M Automated anatomical labeling of activations in SPM using a macroscopic anatomical parcellation of the MNI MRI single-subject brain NeuroImage 2002 15 273 289 10.1006/nimg.2001.0978 11771995

[B36] He Y Chen ZJ Evans AC Small-world anatomical networks in the human brain revealed by cortical thickness from MRI Cerebral cortex 2007 17 2407 2419 17204824 10.1093/cercor/bhl149

[B37] van Wijk BC Stam CJ Daffertshofer A Comparing brain networks of different size and connectivity density using graph theory PLoS One 2010 5 e13701 10.1371/journal.pone.0013701 21060892 PMC2965659

[B38] Rubinov M Sporns O Complex network measures of brain connectivity: uses and interpretations NeuroImage 2010 52 1059 1069 10.1016/j.neuroimage.2009.10.003 19819337

[B39] Maslov S Sneppen K Specificity and stability in topology of protein networks Science 2002 296 910 913 10.1126/science.1065103 11988575

[B40] Milo R Shen-Orr S Itzkovitz S Kashtan N Chklovskii D Alon U Network motifs: simple building blocks of complex networks Science 2002 298 824 827 10.1126/science.298.5594.824 12399590

[B41] Supekar K Menon V Rubin D Musen M Greicius MD Network analysis of intrinsic functional brain connectivity in Alzheimer's disease PLoS Comput Biol 2008 4 e1000100 10.1371/journal.pcbi.1000100 18584043 PMC2435273

[B42] Sailer M Fischl B Salat D Tempelmann C Schoenfeld MA Busa E Bodammer N Heinze HJ Dale A Focal thinning of the cerebral cortex in multiple sclerosis Brain 2003 126 1734 1744 10.1093/brain/awg175 12805100

[B43] Sepulcre J Goni J Masdeu JC Bejarano B Velez N Toledo JB Villoslada P Contribution of white matter lesions to gray matter atrophy in multiple sclerosis Arch Neurol 2009 66 173 179 10.1001/archneurol.2008.562 19204153

[B44] Dietrich J Han R Yang Y Mayer-Proschel M Noble M CNS progenitor cells and oligodendrocytes are targets of chemotherapeutic agents in vitro and in vivo J Biol 2006 5 22 10.1186/jbiol50 17125495 PMC2000477

[B45] Seigers R Schagen SB Beerling W Boogerd W van Tellingen O van Dam FS Koolhaas JM Buwalda B Long-lasting suppression of hippocampal cell proliferation and impaired cognitive performance by methotrexate in the rat Behav Brain Res 2008 186 168 175 10.1016/j.bbr.2007.08.004 17854921

[B46] Winocur G Vardy J Binns MA Kerr L Tannock I The effects of the anti-cancer drugs, methotrexate and 5-fluorouracil, on cognitive function in mice Pharmacol Biochem Behav 2006 85 66 75 10.1016/j.pbb.2006.07.010 16935324

[B47] Han R Yang YM Dietrich J Luebke A Systemic 5-fluorouracil treatment causes a syndrome of delayed myelin destruction in the central nervous system J Biol 2008 7 4 12 10.1186/jbiol69 18430259 PMC2397490

[B48] Seigers R Schagen SB Coppens CM van der Most PJ van Dam FS Koolhaas JM Buwalda B Methotrexate decreases hippocampal cell proliferation and induces memory deficits in rats Behav Brain Res 2009 201 279 284 10.1016/j.bbr.2009.02.025 19428645

[B49] Sporns O The human connectome: a complex network Annals NewYork Academy Sci 2011 1224 109 25 10.1111/j.1749-6632.2010.05888.x 21251014

[B50] Kesler SR Bennett FC Mahaffey ML Spiegel D Regional brain activation during verbal declarative memory in metastatic breast cancer Clin Cancer Res 2009 15 6665 6673 10.1158/1078-0432.CCR-09-1227 19843664 PMC2859687

[B51] Silverman DH Dy CJ Castellon SA Lai J Pio BS Abraham L Waddell K Petersen L Phelps ME Ganz PA Altered frontocortical, cerebellar, and basal ganglia activity in adjuvant-treated breast cancer survivors 5-10 years after chemotherapy Breast Cancer Res Treat 2007 103 303 311 10.1007/s10549-006-9380-z 17009108

[B52] Samatovicz RA Genetics and brain injury: apolipoprotein E J head trauma rehab 2000 15 869 874 10.1097/00001199-200006000-00002 10785619

[B53] Ahles TA Saykin AJ Noll WW Furstenberg CT Guerin S Cole B Mott LA The relationship of APOE genotype to neuropsychological performance in long-term cancer survivors treated with standard dose chemotherapy Psycho-Oncology 2003 12 612 619 10.1002/pon.742 12923801

[B54] Krabbe KS Reichenberg A Yirmiya R Smed A Pedersen BK Bruunsgaard H Low-dose endotoxemia and human neuropsychological functions Brain Behav Immun 2005 19 453 460 10.1016/j.bbi.2005.04.010 15963684

[B55] Wilson CJ Finch CE Cohen HJ Cytokines and cognition–the case for a head-to-toe inflammatory paradigm J Am Geriatr Soc 2002 50 2041 2056 10.1046/j.1532-5415.2002.50619.x 12473019

[B56] Aalto S Bruck A Laine M Nagren K Rinne JO Frontal and temporal dopamine release during working memory and attention tasks in healthy humans: a positron emission tomography study using the high-affinity dopamine D2 receptor ligand [11 C]FLB 457 J neurosci official j Soc Neurosci 2005 25 2471 2477 10.1523/JNEUROSCI.2097-04.2005 PMC6725173 15758155

[B57] Vehmanen L Elomaa I Blomqvist C Saarto T Tamoxifen treatment after adjuvant chemotherapy has opposite effects on bone mineral density in premenopausal patients depending on menstrual status J Clin Oncol 2006 24 675 680 10.1200/JCO.2005.02.3515 16446340

[B58] Boulware MI Kent BA Frick KM The Impact of Age-Related Ovarian Hormone Loss on Cognitive and Neural Function Curr Top Behav Neurosci 2012 10 165 184 21533680 10.1007/7854_2011_122

[B59] Castellon SA Ganz PA Bower JE Petersen L Abraham L Greendale GA Neurocognitive performance in breast cancer survivors exposed to adjuvant chemotherapy and tamoxifen J Clin Exp Neuropsychol 2004 26 955 969 10.1080/13803390490510905 15742545

[B60] Eberling JL Wu C Tong-Turnbeaugh R Jagust WJ Estrogen- and tamoxifen-associated effects on brain structure and function NeuroImage 2004 21 364 371 10.1016/j.neuroimage.2003.08.037 14741674

[B61] Poreh A The Quantified Process Approach to Neuropsychological Assessment 2006 Taylor and Francis, New York, NY

